# ATP Is Required and Advances Cytokine-Induced Gap Junction Formation in Microglia In Vitro

**DOI:** 10.1155/2013/216402

**Published:** 2013-04-23

**Authors:** Pablo J. Sáez, Kenji F. Shoji, Mauricio A. Retamal, Paloma A. Harcha, Gigliola Ramírez, Jean X. Jiang, Rommy von Bernhardi, Juan C. Sáez

**Affiliations:** ^1^Departamento de Fisiología, Pontificia Universidad Católica de Chile, Alameda 340, 6513677 Santiago, Chile; ^2^Instituto Milenio, Centro Interdisciplinario de Neurociencias de Valparaíso, Pasaje Harrington 287, Playa Ancha, 2360103 Valparaíso, Chile; ^3^Departamento de Fisiología, Facultad de Medicina, Clínica Alemana-Universidad del Desarrollo, Las Condes 12438, 7710162 Santiago, Chile; ^4^Departamento de Neurología, Escuela de Medicina, Pontificia Universidad Católica de Chile, Marcoleta 392, 8330024 Santiago, Chile; ^5^Department of Biochemistry, University of Texas Health Science Center, 7703 Floyd Curl Drive, San Antonio, TX 78229, USA

## Abstract

Microglia are the immune cells in the central nervous system. After injury microglia release bioactive molecules, including cytokines and ATP, which modify the functional state of hemichannels (HCs) and gap junction channels (GJCs), affecting the intercellular communication via extracellular and intracellular compartments, respectively. Here, we studied the role of extracellular ATP and several cytokines as modulators of the functional state of microglial HCs and GJCs using dye uptake and dye coupling techniques, respectively. In microglia and the microglia cell line EOC20, ATP advanced the TNF-*α*/IFN-*γ*-induced dye coupling, probably through the induction of IL-1*β* release. Moreover, TNF-*α*/IFN-*γ*, but not TNF-*α* plus ATP, increased dye uptake in EOC20 cells. Blockade of Cx43 and Panx1 HCs prevented dye coupling induced by TNF-*α*/IFN-*γ*, but not TNF-*α* plus ATP. In addition, IL-6 prevented the induction of dye coupling and HC activity induced by TNF-*α*/IFN-*γ* in EOC20 cells. Our data support the notion that extracellular ATP affects the cellular communication between microglia through autocrine and paracrine mechanisms, which might affect the timing of immune response under neuroinflammatory conditions.

## 1. Introduction

Microglia are the major immune effectors in the central nervous system (CNS). Under resting conditions, surveillance microglia have a ramified morphology and monitor their local microenvironment [1, 2]. However, microglia can rapidly become activated in response to diverse stimuli and danger signals, such as ATP or bacterial lipopolysaccharide (LPS) [1–3]. Consistently, microglia are activated in neuroinflammatory conditions and are a common hallmark in many neurodegenerative diseases [1, 2, 4]. 

 Microglial cell activation includes morphological changes, proliferation, recruitment to the site of injury, and expression of specific proteins including MHC II molecules and cell adhesion molecules [1, 2]. Activated microglia also release cytokines, including TNF-*α*, IL-1*β*, IL-6, IFN-*γ*, and other soluble molecules, such as glutamate and ATP [5–9]. Many of these pro-inflammatory molecules act in an autocrine manner and show synergism, increasing the activation of microglia [10–12]. 

 Many studies have focused on ATP release mechanisms and the subsequent receptors activation at the CNS, because they promote the release of other pro-inflammatory molecules, such as TNF-*α* and IL-1*β* [13]. These cytokines mediate cell communication and Ca^2+^ signaling among microglia, as well as among microglia and astrocytes [14–16]. Microglia sense extracellular ATP through P2Y and P2X receptors [1]. Under control conditions, microglia express P2X_7_ receptors, which are upregulated as a required step for microglial activation induced by amyloid-*β* peptide [17, 18]. Moreover, activation of microglia with LPS increases the intracellular free Ca^2+^ concentration ([Ca^2+^]_*i*_) and ATP release, through P2X_7_ receptors [17, 19, 20]. Accordingly, cytokines that increase [Ca^2+^]_*i*_ or a calcium ionophore induce microglia activation [21, 22]. These conditions also induce gap junctional communication in primary cultures of rat or mouse microglia [23, 24].

 Gap junction channels (GJCs) communicate the cytoplasm of contacting cells allowing the direct transfer of ions, second messengers, and other molecules including antigen peptides [25]. Each GJC is formed by the serial docking of two hemichannels (HCs), which are composed of six protein subunits called connexins (Cxs) [25]. It is known that resting microglia express Cxs 32, 36, 43, and 45 and after microglia activation some of them form functional GJCs and HCs [23, 24, 26–28]. Recently, another family of proteins termed pannexins (Panxs) has been found to form functional GJCs and HCs [29]. Like Cx HCs, Panx HCs are permeable to ATP and are activated by increased [Ca^2+^]_*i*_ and extracellular ATP via P2 receptors [30–32]. Microglia express functional Panx1 HCs that contribute to ATP-induced migration and glutamate and ATP release promoting neuronal death [33–35]. Under inflammatory conditions, gap junctional communication between cultured astrocytes is reduced, whereas the activity of HCs is increased [35–38]. However, it remains unknown if these opposite changes in GJCs and HCs also occur in microglia, or if extracellular ATP plays a role in this channel-based communication.

In this work, we studied the effect of extracellular ATP on the cytokine-induced gap junctional communication in microglia. To achieve this goal, we used primary cultures of rat microglia and EOC20 cells treated with several cytokines and ATP, either mixed or alone. We propose that TNF-*α*/IFN-*γ* induce gap junctional communication, which might depend on the functional expression of HCs. In addition, we found that extracellular ATP advances the onset of cytokine-induced expression of gap junctional communication, a process that was mediated by IL-1*β* release and inhibited by IL-6.

## 2. Materials and Methods

### 2.1. Materials

Modified Eagle's medium (MEM), Dulbecco's modified Eagle's medium (DMEM), F-12 nutrient mixture, fetal bovine serum (FBS), bovine pancreas DNAse I, and trypsin-EDTA were purchased from Gibco (Auckland, NZ, USA). DMSO, HEPES, H_2_O, LaCl_3_ (La^3+^), ethidium (Etd) bromide, Lucifer yellow dilithium salt (LY, MW: 457,25 Da), rhodamine-dextran (RD, MW: 10 kDa), adenosine 5′-triphosphate periodate oxidized sodium salt (oATP), ATP disodium salt, probenecid (Pbc), recombinant mouse TNF-*α*, recombinant mouse IL-1*β*, recombinant mouse IFN-*γ*, recombinant mouse IL-6, and Ponceau S red were purchased from Sigma-Aldrich (St Louis, MO, USA). Interleukin-1 receptor antagonist (IL-1ra) was from R&D (USA). BAPTA-AM was purchased from Molecular Probes (Eugene, Oregon, USA). Penicillin, and streptomycin were obtained from Invitrogen (Carlsbad, CA, USA). D(+)-glucose, sodium hydrogen carbonate (NaHCO_3_) were purchased from Merck (Darmstadt, Germany). ^10^Panx1 mimetic peptide (sequence WRQAAFVDSY) was purchased from SBS Biotech (Beijing, China). Purified rat anti-mouse CD16/CD32 (mouse BD Fc-block) was purchased from BD Pharmingen (San José, CA, USA). F(ab′)_2_ fragments of a previously characterized polyclonal rabbit anti-Panx1 serum used [39, 40]. The F(ab′)_2_ fragments of affinity IgGs purified from the anti-Panx1 serum were prepared as previously described [41]. Anti-Cx43 monoclonal antibody was obtained from BD Biosciences (Minneapolis, MN, USA). Cy2 conjugated goat anti-rabbit and Cy3 conjugated goat anti-mouse antibodies were purchased from Jackson Immunoresearch Laboratories Inc. (Indianapolis, IN, USA). EDTA solution, Halt protease inhibitor single-use cocktail, and M-PER mammalian protein extraction reagent were purchased from Thermo Scientific (Rockford, IL, USA). Mount solution fluoromount G was purchased from Electron Microscopy Sciences (Washington, PA, USA). Images were examined with a confocal laser-scanning microscope which was Olympus Fluoview FV1000 (Tokio, Japan). Cx43^(E2)^ is a rabbit polyclonal antibody that recognizes amino acid residues located at the second extracellular loop of Cx43 and blocks specifically Cx43 HCs [42].

Bio-Rad protein assay was purchased from Bio-Rad Laboratories (Richmond, CA, USA). SuperSignal kit for enhanced chemiluminescence detection and anti-rabbit antibody conjugated to horseradish peroxidase were purchased from Pierce (Rockford, IL, USA). EOC20 and LADMAC cells were obtained from ATCC (Manassas, VA, USA). Tissue culture flasks (25 and 75 cm^2^) 60 mm and 100 mm tissue culture dishes were purchased from Sarstedt (Newton, NC, USA). Twenty four-well plastic dishes were purchased from Nunclon (Roskilde, Denmark).

### 2.2. Cell Cultures

#### 2.2.1. Rat Microglia

Primary cultures of microglia were prepared from neocortex of newborn Sprague Dawley rats, as previously described [23, 24]. Briefly, meninges were carefully peeled off and cortices were dissected and minced in small pieces. After incubation in Ca^2+^-free PBS containing trypsin (0.5%) and EDTA (5 mM) at 37°C for 30 min, tissue was triturated in presence of DNAse using a Pasteur pipette. Dissociated cells were pelleted and resuspended in MEM medium supplemented with 10% FBS, 100 units/mL penicillin and 50 *μ*g/mL streptomycin sulfate and plated on plastic culture flasks (Sarstedt). Confluent glial cell mixed cultures were deprived of fresh medium for two weeks to induce microglial cell proliferation. Finally, microglia were harvested from glial cell mixed cultures by differential adhesion and seeded on glass coverslips.

#### 2.2.2. EOC20 Cells

EOC20 cells are a murine microglial cell line derived from C3H/HeJ mice, which secrete cytokines and present antigens as primary microglia [43]. EOC20 cells were maintained according to ATCC recommendations, using DMEM supplemented with 10% FBS and 20% LADMAC conditioned medium (see below). The medium was partially changed twice a week and completely changed once a week until the culture reached confluence. Cells were detached with trypsin-EDTA for 2 min and mechanical stress, and EOC20 cells were seeded on glass coverslips or tissue culture dishes. Since rat microglia were detached by shaking using an orbital shaker, some experiments were performed with EOC20 cells detached with the same methods of purification used for primary microglia cultures. No differences were observed in the induction of dye coupling between EOC20 cells obtained by the different purification methods (data not shown).

#### 2.2.3. LADMAC Conditioned Medium

The conditioned medium was obtained from LADMAC cells, which are myeloid cells derived from murine bone marrow cells. LADMAC cells are nonadherent cells that secrete colony-stimulating-factor-1 (CSF-1) which stimulates cell division in EOC20 cells [43, 44]. LADMAC cell cultures were maintained in culture in MEM supplemented with 10% FBS during two weeks. Fresh medium was added every two days duplicating the previous amount of medium. After two weeks in culture, the cell suspension was centrifuged and the CSF-1-containing supernatant was filtered, aliquoted, and stored at −20°C until use.

### 2.3. Dye Transfer Technique

The transference of fluorescent dyes between adjacent cells has been used to monitor the functional state of GJCs in microglia [23, 24, 27]. We tested the intercellular transference of LY using RD as a negative control. Dyes (5% w/v in 150 mM LiCl) were microinjected by applying current to microglia seeded on glass coverslips (8 × 10^5^ cells/well, in a 24-multiwell dish) through glass microelectrodes until the impaled cells were fluorescent. Cultures were maintained in F-12 medium supplemented with HEPES and observed with an inverted microscope equipped with Xenon arc lamp illumination and a Nikon B filter (excitation wavelength, 450–490 nm; emission wavelength, above 520 nm). Dye transfer was scored at 2 min injection. The incidence of dye coupling (IDC) was calculated as the percentage of injected cells with dye transfer to one or more neighboring cells by the total number of cells microinjected in each experiment. At least 10 cells were microinjected in each assay. Since cytokine treatments induced HC activity and because that dye uptake from leaking microelectrodes could affect the measurement of fluorescent cells, we use 200 *μ*M La^3+^ in the recording solution. However, no significant differences were observed compared to recording solution without La^3+^ (data not shown).

### 2.4. Dye Uptake, Ca^2+^ Signal Imaging, and Time-Lapse Fluorescence Imaging

To evaluate dye uptake, cells seeded on glass coverslips (8 × 10^5^ cells/mL) were exposed to 5 *μ*M ethidium (Etd) bromide with Locke's saline solution (in mM: 154 NaCl; 5.4 KCl; 2.3 CaCl_2_; 1 mM MgCl_2_; 5 mM glucose; 5 mM HEPES; pH: 7.42) and examined by epifluorescence. Nuclei fluorescence was recorded in regions of interest consisting of 30 different cells per field with a water immersion Olympus 51W1I upright microscope (Melville, NY, USA), as described [45]. The calculation of slope change regression lines was fitted to points before and after treatments using Microsoft (Seattle, WA, USA) Excel. In ATP-induced dye uptake experiments, 500 *μ*M ATP was added to recording solution after 5 min of basal dye uptake.

To evaluate Ca^2+^ signals, EOC20 cells under control conditions or after treatment were maintained as mentioned above but were loaded for 30 min with 5 *μ*M Fura-2 AM in DMEM medium without serum at 37°C. Loaded cells were washed twice with Locke's solution and time-measurements were performed with an Olympus 51W1I microscope. The acquisition of 340 and 380 nm excitation wavelengths was every 3 s. Regions of interest consisted in 30 cells per field and analysis were performed using METAFLUOR software.

### 2.5. Western Blot

Confluent microglia cultures grown in 60 mm culture dishes (2.4 × 10^6^ cells) were gently rinsed twice with cold PBS at 4°C, pH 7.4 and harvested by scraping with a rubber policeman in a solution containing 5 mM EDTA, Halt, and M-PER protein extraction cocktail according to the manufacturer's instructions. The cellular suspension was sonicated on ice. Proteins were measured in aliquots of cell lysates using the Bio-Rad protein assay. Aliquots of cell lysates (50 *μ*g of protein) were resuspended in Laemli's sample buffer and separated in an 8% sodium dodecyl sulfate polyacrylamide gel electrophoresis and electrotransferred to nitrocellulose sheets as previously described [24]. Loading equivalences were confirmed by protein staining with Ponceau S red (2% w/v in 30% trichloroacetic acid). Nonspecific protein binding was blocked by incubation of nitrocellulose sheets in 5% nonfat milk in PBS for 1 h at room temperature prior to overnight incubation with corresponding antibodies at 4°C. After several washes with PBS, blots were incubated with the secondary antibody conjugated to horseradish peroxidase for 45 min at room temperature. Immunoreactivity was detected by enhanced chemiluminescence using the SuperSignal kit according to the manufacturer's instructions.

### 2.6. Immunofluorescence

Microglia cultured on glass cover slips were fixed with 4% formaldehyde at room temperature for 30 min and washed twice with PBS. A blocking solution containing 1% IgG free BSA, 50 mM NH_4_Cl, and 0.05% Triton X-100 in PBS was used to permeabilize and block unspecific reactive sites. Fc receptors were masked by incubating samples to a solution containing Fc-Block (1 : 100) for 45 min at room temperature. Panx1 and Cx43 were detected with a rabbit polyclonal anti-Panx1 F(ab′)_2_ fragments [40] and an anti-Cx43 monoclonal antibody, properly diluted with blocking solution, respectively. Cy2 conjugated goat anti-rabbit (1 : 300) and Cy3 conjugated goat anti-mouse IgGF(ab′)_2_ Igs fragments for 30 min at room temperature were used to detect bound primary antibody. Fluoromount G (Electron Microscopy Sciences, Washington, PA, USA) was used as an antifade solution to mount samples. Images were examined with a confocal laser-scanning microscope (Olympus, Fluoview FV1000, Tokio, Japan).

### 2.7. IL-1*β* ELISA

The level of IL-1*β* present in the conditioned media of EOC20 cells was evaluated with the IL-1*β* ELISA Ready.Set-Go! (e-Bioscience, San Diego, CA, USA), for performing quantitative enzyme linked immunosorbent assays (ELISA). It has a sensitivity of 8 pg/mL. Standard curve consisted of twofold serial dilutions of the recombinant cytokine. In brief, a 96-well, flat bottom, ELISA-plate (MICROLON, Greiner Bio-One) was coated with capture antibody in coating buffer overnight at 4°C. The plate was washed 5 times with PBS-0.05% Tween-20 in ELx50 Biokit, a 96-well bioelisa washer, and Rhe plate was blocked with 200 *μ*L of assay diluent at room temperature for 1 h, washed as mentioned, and 100 *μ*L of standard IL1*β* and samples were incubated at 4°C overnight. Then, the plate was washed and 100 *μ*L of detection antibody for IL-1*β* was added and incubated at room temperature by 1 h, washed 5 times, incubated with 100 *μ*L Avidin-HRP at room temperature for 30 min, washed 7 times, added 100 *μ*L substrate solution, and stopped the reaction with 50 *μ*L of 1 M H_3_PO_4_. The plate was read at 450 nm, with reference at 570 nm.

### 2.8. Treatments

Microglia were seeded 48 h before dye transfer, dye uptake, or immunofluorescence experiments in 24-well plastic dish containing 500 *μ*L of culture medium. For Western blot experiments, cells were seeded in 60 mm plastic dishes in 3 mL of culture medium. After 48 h under control conditions microglia were treated with 1 mM ATP or 1 ng/mL TNF-*α*, IFN-*γ*, IL-1*β* either alone or mixed. Cytokines were added simultaneously and ATP was added 2 h before measurement and is referred as cytokine(s) plus ATP. Treatment with 1, 10, or 50 ng/mL IL-6, 20 ng/mL IL-1ra, 300 *μ*M oATP, 200 *μ*M La^3+^, 1 : 500 Cx43^(E2)^ antibody or 200 *μ*M ^10^Panx1 was simultaneous to cytokine treatment. We used 50 *μ*M of *β*-GA for acute GJCs blocking (Figure S1, see Supplementary Materials available online at http://dx.doi.org/10.1155/2013/216402). To avoid disruption of cell adhesion with BAPTA, the medium was replaced with culture medium of parallel cultures treated at the same time to maintain the soluble factor released from microglia.

### 2.9. Statistical Analysis

Data are presented as mean ± SEM, as percentage of the control condition; *n* represents the number of independent experiments. For statistical analysis, each treatment was compared with its respective control and significance was determined using one-way ANOVA followed by Dunn's test comparing all treatments against the control condition. To observe differences between microglia and EOC20 cells responses we used a two-way ANOVA.

## 3. Results

### 3.1. The Onset of the Cytokine-Induced Increase in Gap Junctional Communication in Cultured Microglia Is Advanced by ATP and Delayed by IFN-*γ*

Calcium ionophore and pro-inflammatory molecules promote a transient expression of functional GJCs in microglia [23, 24, 27]. Since extracellular ATP, TNF-*α*, and IFN-*γ* play a relevant role in microglial cell responses [3, 7, 46] and affect the [Ca^2+^]_*i*_ [47–49], we decided to evaluate if these compounds affect the intercellular communication via GJCs in both primary cultures of rat microglia and EOC20 cells.

**Figure 1:**
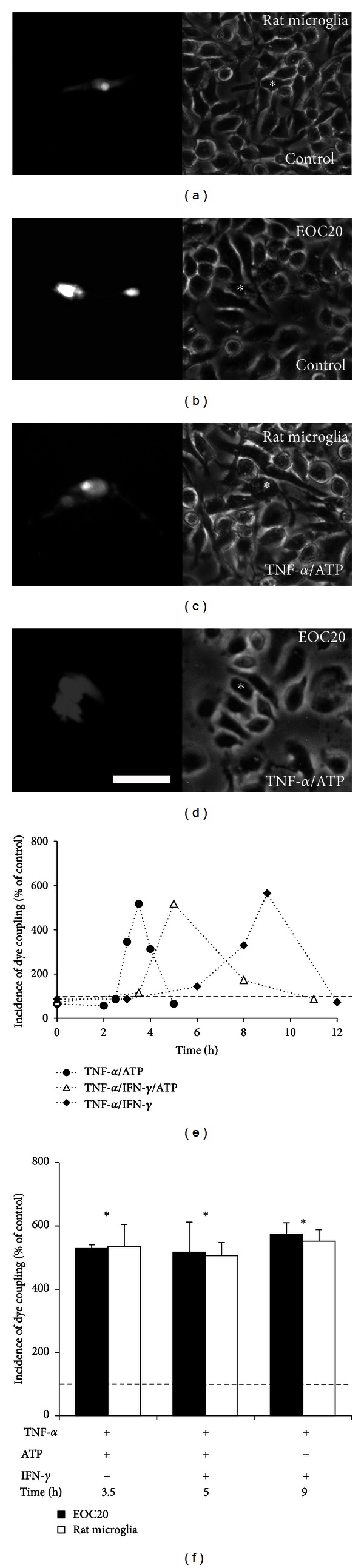
ATP advances the onset of TNF-*α*/IFN-*γ*-induced dye coupling. (a)–(d) Dye transfer was evaluated 2 min after Lucifer yellow (LY) microinjection in a single cell (indicated with an asterisk). Representative pictures of LY transfer in rat microglia (a), (c) or EOC20 cells (b), (d) under control condition or after TNF-*α* plus ATP (TNF-*α*/ATP) treatment for 3.5 h, as indicated. Phase contrasts of each micrograph are shown at the right panels. Scale bar: 20 *μ*m. (e) Time course of the incidence of dye coupling (IDC) as percentage of IDC in EOC20 cells under control conditions (dashed line) or after treatment with TNF-*α* plus ATP (black circles), TNF-*α*/IFN-*γ* plus ATP (white triangles), or TNF-*α*/IFN-*γ* (black diamonds). Each point corresponds to the mean of 3 independent experiments. (f) Graph showing the maximum values of IDC after treatment with TNF-*α* plus ATP for 3.5 h, TNF-*α*/IFN-*γ* plus ATP for 5 h, or TNF-*α*/IFN-*γ* for 9 h. *P < 0.05 versus control condition. Each bar represents the mean ± SEM, *n* = 6. No significant differences were observed when comparing microglia and EOC20 cells responses to different treatment in dye transfer assays. Concentrations: 1 ng/mL TNF-*α*; 1 mM ATP; 1 ng/mL IFN-*γ*.

After 48 h of subculture under control conditions, microglia were treated as indicated in Methods (Figure S1a). Both cell types presented rather homogeneous morphological features (Figures 1(a) and 1(b)) and very low incidence of Lucifer yellow (LY) transfer to neighboring cells (Figures 1(a) and 1(b)). Under these conditions, the incidence of dye coupling (I.D.C) remained low for up to 12 h of culture in both cell types (Figure 1, Supporting information Table S1). In addition, intercellular transfer of rhodamine-dextran (RD, ~10 kDa), which due to its high molecular weight cannot permeate through GJCs, was not observed (Figure S2a). This result indicates that intercellular LY transfer ocurred via GJCs and not through other cell-cell communication pathway, such as cytoplasmic bridges. Moreover, microglia treated either with 1 mM ATP, 1 ng/mL TNF-*α*, 1 ng/mL IFN-*γ*, or 1 ng/mL IL-1*β* showed only a slight increase in IDC, which was not statistically different from that of control cells (*P* > 0.05: Supporting information Table S1). However, treatment with mixes of these molecules during different time periods caused a significant and transient increase in IDC; the dye transfer data is expressed as percentage of the corresponding control condition (Figures 1(e) and 1(f)). In both cell types, treatment with 1 ng/mL TNF-*α* plus 1 ng/mL IFN-*γ* (from now and on referred as TNF-*α*/IFN-*γ*) increased the IDC, reaching a maximum response at around 9 h after treatment (IDC in EOC20 cells: 574 ± 36% of control; rat microglia, 552 ± 36% of control; Mean ± SEM; *n* = 5) as previously described [23].

We also studied if extracellular ATP affects TNF-*α*/IFN-*γ*-induced dye coupling. To this end, cells were treated with these cytokines and then exposed to ATP for 2 h. In both cell types, treatment with TNF-*α*/IFN-*γ* plus ATP induced a transient increase in IDC, which was maximal at around 5 h (EOC20 cells: 517 ± 94% of control; rat microglia: 506 ± 42% of control, *n* = 5). The amplitude and duration (magnitude) of the response was similar to that induced by TNF-*α*/IFN-*γ*, but occurred 4 h earlier (Figure 1(e)). 

Since IFN-*γ* potentiates TNF-*α*-induced dye coupling in antigen presenting cells, including dendritic cells, microglia and monocytes/macrophages [23, 50, 51], we tested whether ATP induces a similar effect on microglia. In agreement with this possibility, cells treated with TNF-*α* plus ATP showed maximal IDC with similar amplitude (EOC20 cells: 529 ± 12% of control; rat microglia: 534 ± 70% of control; *n* = 6; Figure 1) to that induced by TNF-*α*/IFN-*γ* plus ATP, but occurred 1.5 h earlier (at ~3.5 h versus 5 h; Figures 1(e) and 1(f)). As mentioned before intercellular transfer of LY was enhanced in primary microglia or EOC20 cells treated with TNF-*α* plus ATP (Figures 1(c) and 1(d)). However, intercellular transfer of RD was not observed, ruling out the formation of cytoplasmic bridges or vesicular mediated dye transfer in each condition (Figure S2). Microglia treated with IFN-*γ* plus ATP did not increase dye coupling at 3.5 h (EOC20 cells: 167 ± 97% of control; rat microglia: 210.8 ± 51.3% of control) or other times (2 and 5 h, data not shown).

### 3.2. The Increase of Gap Junctional Communication Induced by TNF-*α* Plus ATP Requires an Increase of [Ca^2+^]_*i*_ via Activation of P2X Receptors and Is Prevented by IL-6

**Figure 2:**
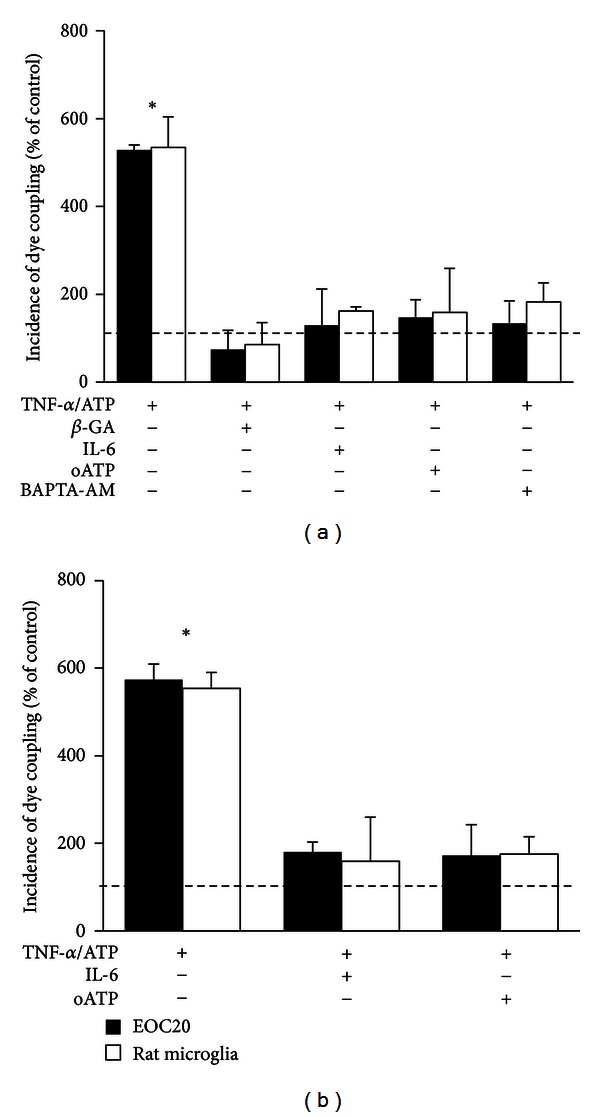
IL-6, intracellular calcium chelation, and P2X inhibition prevent the induction of gap junctional communication promoted by TNF-*α* plus ATP or TNF-*α*/IFN-*γ*. (a) Graph showing the effect of acutely applied 50 *μ*M 18-*β*-glycyrrhetinic acid (*β*-GA) or pretreatment with 10 ng/mL interleukin-6 (IL-6), 300 *μ*M oxidized ATP (oATP), or 10 *μ*M BAPTA-AM on the incidence of dye coupling (IDC) of microglia treated for 3.5 h with TNF-*α* plus ATP. (b) Graph showing the effect of 50 ng/mL IL-6 or 300 *μ*M oATP over the IDC of microglia treated for 9 h with TNF-*α*/IFN-*γ*. Data is expressed as a percentage of IDC under control conditions (dashed line). *P < 0.05 versus control condition. Each bar represents the mean ± SEM, *n* = 5. No significant differences were observed when comparing microglia and EOC20 cells responses to different treatment in dye transfer assays.

Eugenín et al. (2001) described that dye coupling between microglia treated for 9 h with TNF-*α*/IFN-*γ* is inhibited by *β*-GA. In EOC20 cells treated with TNF-*α*/IFN-*γ*, we observed a similar acute blockade with *β*-GA (data not shown). In addition, application of 50 *μ*M *β*-GA for 5 min completely abolished dye coupling induced by TNF-*α* plus ATP (IDC in EOC20 cells: 74 ± 44% of control; rat microglia: 86 ± 50% of control; *n* = 5; Figure 2(a)).

Since microglia treated with purinergic agonists release IL-6 [52], and this cytokine prevents the increase of dye coupling induced by TNF-*α*/IL-1*β* in dendritic cells [50], we decided to test if IL-6 prevents induction of dye coupling in microglia treated with TNF-*α* plus ATP. In cell cultures treated simultaneously with 10 ng/mL IL-6 plus TNF-*α* and then treated with ATP for 3.5 h, the IDC was low (EOC20 cells: 130 ± 83% of control; rat microglia: 162 ± 10% of control; *n* = 4) similar to the results obtained under control conditions (Figure 2(a)). Similarly, the TNF-*α*/IFN-*γ*-induced dye coupling was prevented by IL-6 (Figure 2(b)). This inhibitory effect was IL-6 concentration-dependent (1, 10, and 50 ng/mL, data not shown). The maximal effect was induced by 50 ng/mL IL-6 (EOC20: 180 ± 23% of control; rat microglia: 159 ± 100% of control; *n* = 4; Figure 2(b)).

Since microglia express several P2X and P2Y receptors [3], the possible involvement of purinergic receptors in the TNF-*α*/IFN-*γ*-induced dye coupling in microglia treated with oxidized-ATP (oATP), an inhibitor of P2X receptors [53], was studied. Coapplication of 300 *μ*M oATP prevented dye transfer induced by TNF-*α* plus ATP (IDC in EOC20 cells: 147 ± 41% of control; rat microglia: 159 ± 100% of control; *n* = 5; Figure 2(a)) or by TNF-*α*/IFN-*γ* (IDC in EOC20: 172 ± 70% of control; rat microglia: 176 ± 40% of control; *n* = 5; Figure 2(b)). Moreover, cells treated with TNF-*α* plus 1 mM ADP, a P2Y agonist [53], for 3.5 h did not show changes in dye coupling (IDC in EOC20 cells: 168 ± 84% of control, *n* = 3), suggesting that P2Y receptors are not involved in ATP-induced gap junctional communication in microglia.

Since activation of P2 receptors promotes a rise in [Ca^2+^]_*i*_ in microglia [54], we tested if this response was related to the increase in dye coupling induced by TNF-*α* plus ATP. Cells were loaded with BAPTA, a Ca^2+^ chelator, and then washed and the extracellular medium was replaced with conditioned medium of cultures treated in parallel with TNF-*α* for 90 min to maintain the culture conditions as before loading with BAPTA. In these cells, treatment with TNF-*α* plus ATP did not increase dye coupling (IDC in EOC20 cells: 134 ± 51% of control; rat microglia: 183 ± 44% of control; *n* = 5; Figure 2(a)). In addition, we observed that EOC20 cells treated with TNF-*α* plus ATP present increased Ca^2+^ signal, compared to cells under control conditions (Figure S3a). Interestingly, IL-6 prevented this rise in the Ca^2+^ signal (Figure S3b), suggesting that IL-6 might regulate the purinergic signaling in EOC20 cells.

### 3.3. IL-1*β* Released by Activated Microglia Mediates the TNF-*α*/IFN-*γ*-Induced Dye Coupling in EOC20 Cells

**Figure 3:**
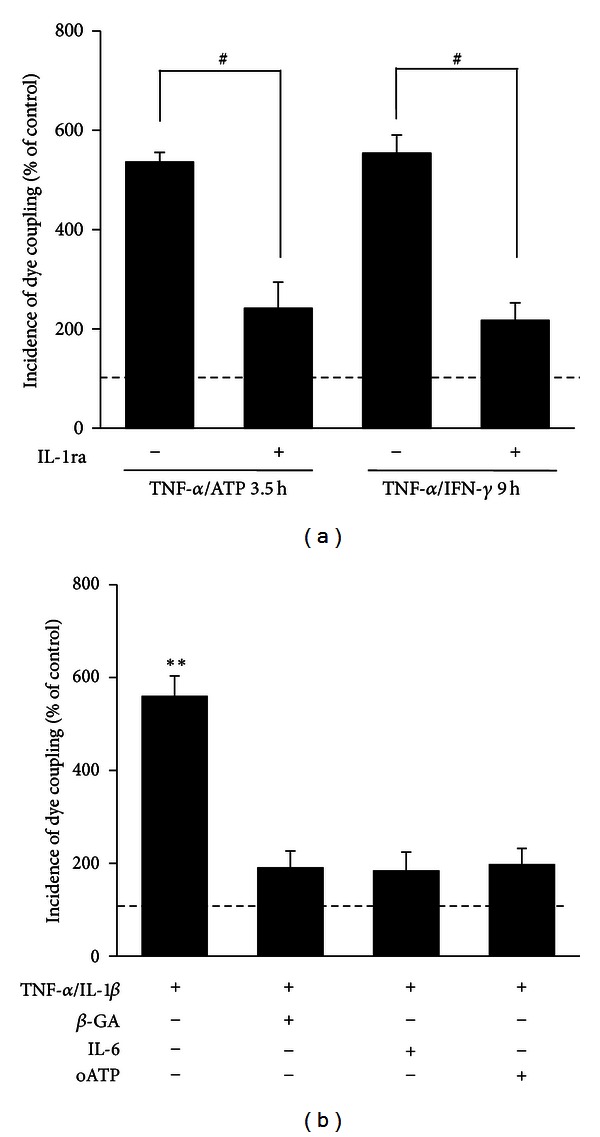
IL-1*β* mediates gap junctional communication induced by pro-inflammatory molecules. (a) Effect of 20 ng/mL IL-1 receptor agonist (IL-1ra) over LY transfer in EOC20 cells treated with TNF-*α* plus ATP for 3.5 h or with TNF-*α*/IFN-*γ* for 9 h. ^#^ *P* < 0.05 between indicated treatments. Each bar represents the mean ± SEM, *n* = 4. (b) The effect of 50 *μ*M 18-*β*-glycyrrhetinic acid (*β*-GA) acutely applied or treatment with 10 ng/mL interleukin-6 (IL-6) or 300 *μ*M oxidized ATP (oATP) over LY transfer in EOC20 cells treated with TNF-*α*/IL-1*β* for 9 h is also shown. Each bar represents the mean ± SEM, *n* = 4, and corresponds to the percentage of incidence of dye coupling under control conditions (dashed line). *P < 0.05, **P < 0.01 versus control condition.

Since activated microglia release IL-1*β* and its natural antagonist IL-1ra [7, 55], we studied possible involvement of these molecules in the transient increase in dye coupling induced by TNF-*α* plus ATP or TNF-*α*/IFN-*γ*. Coapplication of 20 ng/mL IL-1ra significantly prevented the increase in IDC induced by TNF-*α* plus ATP (in EOC20 cells: 217 ± 36% of control, *n* = 4) or TNF-*α*/IFN-*γ* (in EOC20 cells: 241 ± 53% of control, *n* = 4; Figure 3(a)). Moreover, EOC20 cells showed an increase in IL-1*β* release after TNF-*α* plus ATP or TNF-*α*/IFN-*γ* stimulation, which was partially prevented by IL-6 (Figure S4). Consistent with the involvement of IL-1*β* in the above dye coupling response induced by both pro-inflammatory molecules, exogenous application of 1 ng/mL IL-1*β* plus TNF-*α* induced a similar response than that elicited by TNF-*α* plus ATP or TNF-*α*/IFN-*γ* (Figure 3(b)). EOC20 cells treated with TNF-*α*/IL-1*β* showed a transient increase in dye coupling (data not shown), reaching a maximal IDC at ~9 h of treatment (EOC20 cells: 560 ± 43% of control, *n* = 4; Figure 3(b)). The TNF-*α*/IL-1*β*-induced dye coupling was drastically reduced by the acute application of 50 *μ*M *β*-GA (IDC in EOC20 cells: 192 ± 35% of control, *n* = 4) and prevented by 10 ng/mL IL-6 (in EOC20 cells: 185 ± 40% of control, *n* = 4) or 300 *μ*M oATP (in EOC20 cells: 198 ± 34% of control, *n* = 4) coapplied with the two cytokines (Figure 3(b)). However, treatment with IL-1*β* did not increase dye coupling in EOC20 cells (data not shown).

### 3.4. TNF-*α*/IFN-*γ* but Not TNF-*α* Plus ATP Increases Plasma Membrane Permeability in EOC20 Cells

**Figure 4:**
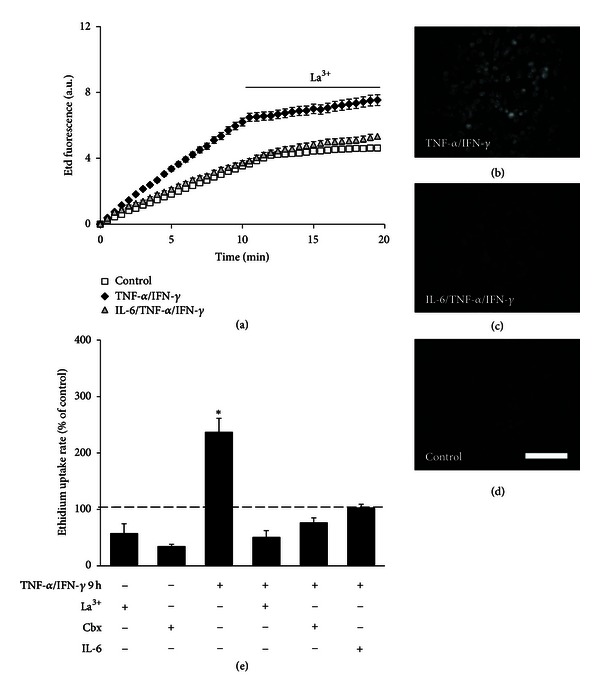
TNF-*α*/IFN-*γ* induces membrane permeabilization in EOC20 cells. (a) Time-lapse measurements of ethidium (Etd) uptake in EOC20 cells under control conditions (white squares), or after treatment with TNF-*α*/IFN-*γ* (black diamonds) or IL-6/TNF-*α*/IFN-*γ* (gray triangles) for 9 h. Each value represents the mean ± SEM of 30 cells. After 10 min of basal uptake, 200 *μ*M La^3+^ was applied to the bath. (b)–(d) Representative fluorescence micrographs of Etd uptake after 10 min of Etd uptake under indicated treatments, previous to La^3+^ application. Scale bar: 100 *μ*m. (e) Graph showing the acute effect of 200 *μ*M La^3+^, 10 *μ*M carbenoxolone (Cbx), or pretreatment with 50 ng/mL of interleukin-6 (IL-6) Etd uptake rate expressed as percentage of control conditions (dashed line) in EOC20 cells treated with TNF-*α*/IFN-*γ* for 9 h. Each bar corresponds to the mean ± SEM, *n* = 5. *P < 0.05 versus control condition.

Astrocytes treated with TNF-*α*/IL-1*β* for 24 h [38] and microglia treated with LPS (or TNF-*α*) for 24 h showed an increased HC activity [28, 35, 56–58]. Using the ethidium (Etd) uptake assay to evaluate the functional state of HCs located at the cell surface [38, 59], we studied if TNF-*α* or ATP affects the membrane permeability of microglia cells. In EOC20 cells, Etd uptake evaluated with time-lapse measurements showed no significant differences after treatment with TNF-*α* plus ATP as compared to untreated cells (Figure S5). In control conditions, Etd uptake was partially blocked by 200 *μ*M La^3+^ (after La^3+^: 45 ± 11% of control, *n* = 5), a Cx HC blocker that does not affect Panx HCs [31] and by 10 *μ*M carbenoxolone (Cbx) (after Cbx: 36 ± 15% of control, *n* = 5), which at this concentration inhibits mainly Panx HCs [60]. A slight, but not statistically significant increase in Etd uptake was recorded after 3.5 h treatment with TNF-*α* plus ATP (134 ± 25% of control, *n* = 5) and was inhibited by La^3+^ (after La^3+^: 47 ± 8% of control, *n* = 5) or Cbx (after Cbx: 38 ± 8% of control, *n* = 5), suggesting an upstream cross talk between Cx and Panx HCs. In addition, 10 ng/mL IL-6 did not affect the response induced by TNF-*α* plus ATP treatment for 3.5 h (Etd uptake rate: 141 ± 16% of control, *n* = 5; Figure S5b). In contrast, after treatment with TNF-*α*/IFN-*γ* for 9 h, a statistically significant increase in the Etd uptake rate compared to the control condition was detected (Figure 4). In EOC20 cells cultured for 9 h under control conditions the Etd uptake rate remained low and was partially blocked by La^3+^ (57 ± 17% of control, *n* = 5; Figures 4(a) and 4(e)) or Cbx (34 ± 4% of control, *n* = 5; Figure 4(e)). However, cells treated with TNF-*α*/IFN-*γ* for 9 h showed a prominent increase in Etd uptake (237 ± 25% of control, *n* = 5) that was drastically reduced by La^3+^ (51 ± 12% of control, *n* = 5; Figures 4(a) and 4(e)) or Cbx (76 ± 9% of control, *n* = 5; Figure 4(e)). A similar increase in dye uptake was found after treatment with TNF-*α*/IL-1*β* for 9 h (Etd uptake rate: 197 ± 41% of control, *n* = 3), which was also reduced by La^3+^ (Etd uptake rate: 105 ± 4% of control, *n* = 3). Moreover, coapplication of 50 ng/mL IL-6 with TNF-*α*/IFN-*γ* prevented the Etd uptake rate increase in cells treated just with TNF-*α*/IFN-*γ* (96 ± 67% of control, *n* = 5; Figure 4(e)). In the latter conditions, the Etd uptake rate was slightly reduced by La^3+^ (48 ± 8% of control, *n* = 5). 

### 3.5. Extracellular ATP Increases the Plasma Membrane Permeability in EOC20 Cells

**Figure 5:**
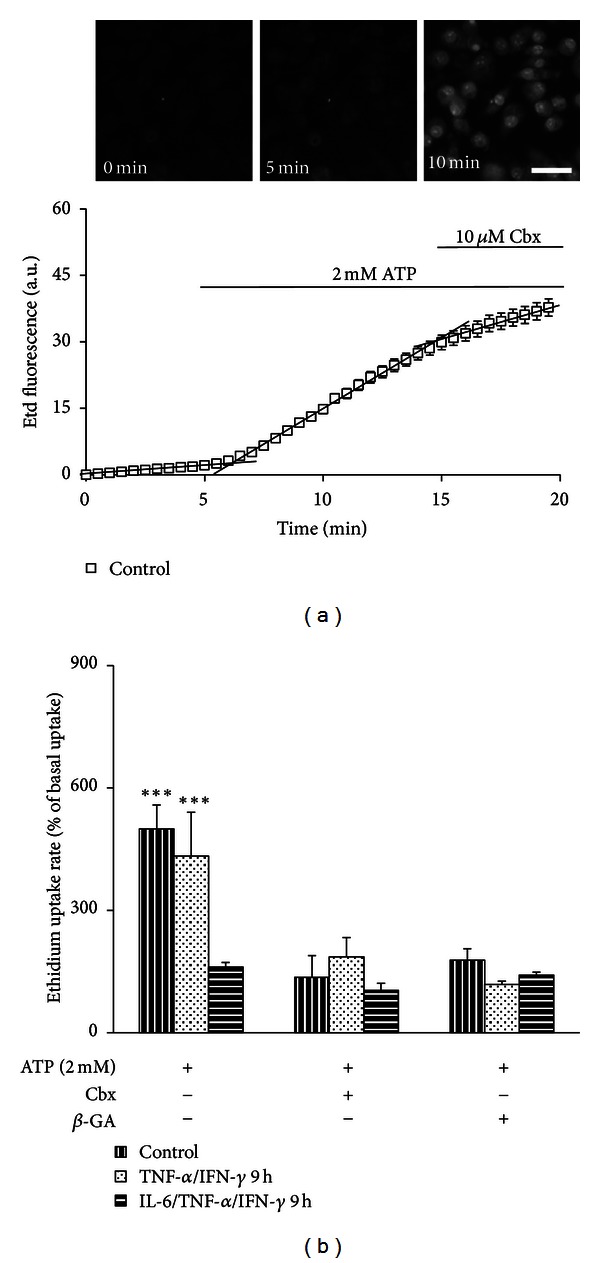
Extracellular ATP increases the cell membrane permeability in EOC20 cells. (a) Fluorescent views of Etd uptake of EOC20 cells cultured under control conditions before (0 min) or after application of 2 mM ATP (5 and 10 min). Scale bar: 25 *μ*m. Time-lapse measurement. After 5 min of basal uptake, 2 mM ATP was added to extracellular solution. At 15 min of recording, 10 *μ*M of carbenoxolone (Cbx) a HC blocker was added to the bath. Black lines denote the slope at different times of Etd uptake. Data represent the mean ± SEM of 30 cells in each of 5 independent experiments. (b) Graph showing the effect of acute application of extracellular ATP in EOC20 cells under control conditions or after treatment with TNF-*α*/IFN-*γ* or with 50 ng/mL IL-6 plus TNF-*α*/IFN-*γ* for 9 h. The effect of acute blockade with 10 *μ*M carbenoxolone (Cbx) or 50 *μ*M 18-*β*-glycyrrhetinic acid (*β*-GA) is also shown. Data was normalized to basal uptake in each condition (dashed line) and represents the mean ± SEM. ***P < 0.001 versus control condition.

Extracellular ATP, in the millimolar range, induces membrane permeabilization in many cell types, including microglia [61, 62]. Similarly, ATP permeabilizes macrophages in a Panx1-dependent way [31]. We tested the effect of 2 mM ATP on Etd uptake in EOC20 cells, as previously observed in macrophages and described by others [31, 63]. A rapid increase in Etd uptake rate (expressed as % of control) was induced by the acute application of 2 mM ATP (529 ± 84% of basal uptake, *n* = 5) to cells cultured for 3.5 h under control conditions (Figures 5(a) and 5(b)). This response was drastically blocked by 10 *μ*M Cbx (218 ± 81% of basal uptake, *n* = 5; Figure 5(a)), as well as by 50 *μ*M *β*-GA, a Cx and Panx HC blocker (128 ± 47% of basal uptake, *n* = 5; Figure 5). In cell cultures treated with TNF-*α* plus ATP for 3.5 h, acute treatment with ATP did not induce a statistically significant increase in Etd uptake (173 ± 17% of basal uptake, *n* = 5, Figure S6a) and was blocked by Cbx (85 ± 16% of basal uptake, *n* = 5) or *β*-GA (102 ± 63% of basal uptake, *n* = 5 Figure S6b). Similarly, cells treated with 10 ng/mL IL-6/TNF-*α* plus ATP showed a small increase in Etd uptake rate after acute application of 2 mM ATP (196 ± 28% of basal uptake, *n* = 5, Figure S6b). This response was blocked by Cbx (85 ± 28% of basal uptake, *n* = 5) or *β*-GA (102 ± 63% of basal uptake, *n* = 5; Figure S6b).

Moreover, EOC20 cells cultured for 9 h under control conditions showed a rapid increase of Etd uptake in response to 2 mM ATP (500 ± 58% of basal uptake, *n* = 5), which was completely blocked by Cbx (136 ± 53% of basal uptake, *n* = 5) or *β*-GA (178 ± 28% of basal uptake, *n* = 5; Figure 5(b)). EOC20 cells treated with TNF-*α*/IFN-*γ* for 9 h exhibited a significant increase in Etd uptake rate after ATP treatment (433 ± 107% of basal uptake, *n* = 5), which was blocked by Cbx (186 ± 47% of basal uptake, *n* = 5) or *β*-GA (118 ± 8% of basal uptake, *n* = 5). In contrast, in EOC20 cells treated for 9 h with 50 ng/mL IL-6 plus TNF-*α*/IFN-*γ*, ATP did not increase Etd uptake (161 ± 11% of basal uptake, *n* = 5), and neither Cbx (104 ± 17% of basal uptake, *n* = 5) nor *β*-GA (141 ± 7% of basal uptake, *n* = 5; Figure 5(b)) affected it.

In addition, cultures treated for 9 h with TNF-*α*/IL-1*β* showed increased Etd uptake rate after ATP application (510 ± 58% of basal uptake, *n* = 5, Figure S7a), which was partially blocked by Cbx (229 ± 32% of basal uptake, *n* = 5, Figure S7a) or *β*-GA (282 ± 35% of basal uptake, *n* = 5). Interestingly, the ATP-induced increase in Etd uptake was almost completely absent in cells pretreated with 10 ng/mL IL-6 plus TNF-*α*/IL-1*β* (243 ± 56% of basal uptake, *n* = 5, Figure S7a) and the activity present was blocked by 10 *μ*M Cbx (210 ± 71% of basal uptake, *n* = 5) or *β*-GA (175 ± 49% of basal uptake, *n* = 5; Figure S7a).

### 3.6. Blockade of Hemichannels Reduces the TNF-*α*/IFN-*γ*-Induced Dye Coupling

**Figure 6:**
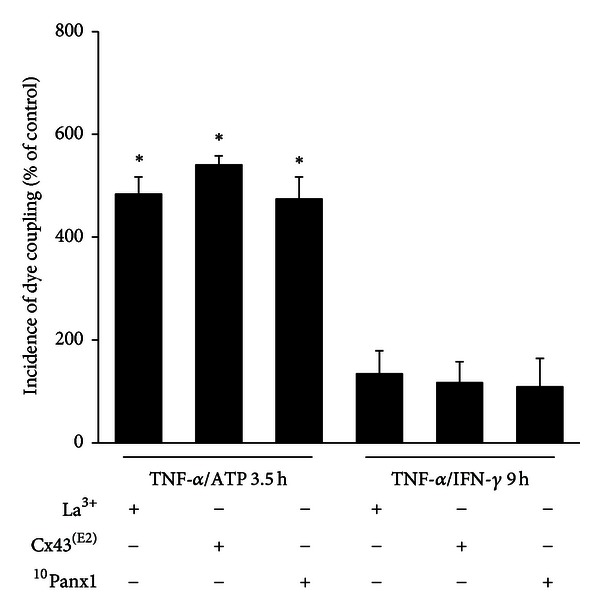
Blockade of HCs prevents upregulation of microglial gap junctional communication induced by TNF-*α*/IFN-*γ*, but not that induced by TNF-*α* plus ATP. Effect on the incidence of dye coupling (IDC) in EOC20 cells treated with TNF-*α* plus ATP for 3.5 h or TNF-*α*/IFN-*γ* for 9 h in presence of HC blockers (200 *μ*M La^3+^, 1 : 500 Cx43^(E2)^ antibody, 200 *μ*M ^10^Panx1). Data is expressed as percentage of IDC under control conditions (dashed line) and corresponds to the mean ± SEM, *n* = 4. *P < 0.05 versus control condition.

Open HCs allow the release of molecules such as ATP and glutamate [35, 56–58, 64] and uptake of small molecules such as glucose [38]. In addition, in other cellular systems, functional Cx46 HCs stimulate formation of GJCs [65]. Thus, we studied the possible contribution of increased HC activity on dye coupling induced by pro-inflammatory molecules in cells incubated with HC blockers. Treatment with 200 *μ*M La^3+^ prevented the TNF-*α*/IFN-*γ*-induced dye coupling recorded as IDC (134 ± 45% of control, *n* = 4; Figure 6). A similar inhibitory effect was induced by the application of 1 : 500 Cx43^(E2)^ antibody (117 ± 41% of control, *n* = 4), a specific Cx43 HC blocker [66], or 200 *μ*M ^10^Panx1 (IDC in EOC20 cells: 109 ± 55% of control, *n* = 4; Figure 6). However, neither irrelevant IgG nor scramble ^10^Panx1 peptide prevented the TNF-*α*/IFN-*γ*-induced dye coupling (data not shown). On the other hand, treatment with La^3+^ (484 ± 34% of control, *n* = 4), Cx43^(E2)^ antibody (540.8 ± 30% of control, *n* = 4) or ^10^Panx1 (474 ± 43% of control, *n* = 4) did not change the dye coupling induced by TNF-*α* plus ATP (Figure 6). 

### 3.7. Pro-Inflammatory Molecules Regulate Cx43 and Panx1 Levels and Distribution in Microglia

Cx32, Cx36, and Cx43 have been detected in cultured microglia [23, 24, 26–28]. However, Cx43 seems to be the main contributor involved in cytokine-induced gap junctional communication, because microglia from Cx43^del/del^ mice do not express functional GJCs in response to TNF-*α*/IFN-*γ* [23]. In addition, expression of Panx1 by microglia has been reported recently [35]. Thus, the distribution and levels of Cx43 and Panx1 during treatments that affect GJC and HC activity were evaluated by immunofluorescence and Western blot analyses. 

**Figure 7:**
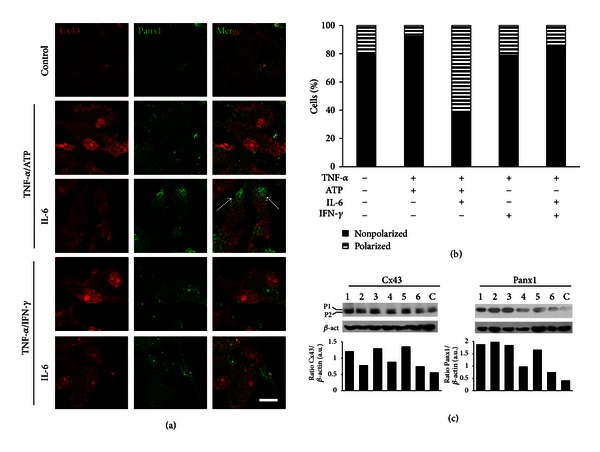
Pro-inflammatory treatments upregulate Cx43 and Panx1 protein levels in microglia. (a) Confocal images show immunoreactivity for Cx43 (red) and Panx1 (green) in primary rat microglia under control conditions of after treatment with TNF-*α* plus ATP for 3.5 h or with TNF-*α*/IFN-*γ* for 9 h, in absence or presence of IL-6 (10 or 50 ng/mL, respectively). Arrows show microglia with segregation of Cx43 and Panx1. Scale bar: 10 *μ*m. (b) Quantification of nonpolarized (black bars) versus polarized (dashed white bars) rat microglia under control conditions or after treatments shown in (a). Data are expressed as a percentage of the total number of cells per field, *n* = 5 (up to 100 cells per field). *P < 0.05 versus control condition. (c) Representative Western blots from 3 independent experiments showing total protein levels of Cx43 and Panx1. Cell lysates were obtained from EOC20 cells under control conditions (lane C) or after the following treatments: TNF-*α* plus ATP (lane 1), IL-6/TNF-*α* plus ATP (lane 2), TNF-*α*/IFN-*γ* (lane 3), IL-6/TNF-*α*/IFN-*γ* (lane 4), TNF-*α*/IL-1*β* (lane 5), and IL-6/TNF-*α*/IL-1*β* (lane 6). Quantitation of Cx43 and Panx1 is shown; *β*-actin was used as a loading control for densitometric analysis.

Under control conditions, rat microglia presented low and heterogeneous Cx43 and Panx1 reactivity (Figure 7(a)). After treatment with TNF-*α* plus ATP (3.5 h) or TNF-*α*/IFN-*γ* (9 h) Cx43 and Panx1 reactivity were higher than in control conditions (Figure 7(a)). However, treatment with IL-6 (10 ng/mL)/TNF-*α* plus ATP or IL-6 (50 ng/mL)/TNF-*α*/IFN-*γ* did not affect the reactivity of Cx43 and Panx1 (Figure 7(a)). Moreover, in cultures treated with IL-6 plus TNF-*α*/ATP a redistribution of Cx43 and Panx1 was observed; these proteins were segregated providing a “cell polarization” appearance, which was quantified (Figure 7(b)). Under control conditions rat microglia exhibited little or no segregation (polarized: 19 ± 6%, *n* = 5) although some cells showed more Cx43 or Panx1 reactivity. Segregation of these proteins was not significantly affected by TNF-*α* plus ATP for 3.5 h (polarized: 8 ± 4%, *n* = 5), but the number of cells with segregation was increased by the simultaneous treatment with IL-6 and TNF-*α* plus ATP (polarized: 61 ± 1%, *n* = 5). However, treatment with TNF-*α*/IFN-*γ* for 9 h did not affect the resting distribution (polarized: 21 ± 6%, *n* = 5) and remained unchanged in cells simultaneously treated with IL-6/TNF-*α*/IFN-*γ* (polarized: 15 ± 4%, *n* = 5). Similar results were found in EOC20 cells treated with TNF-*α*/IL-1*β* for 9 h (Figure S7b).

Protein levels were evaluated in EOC20 cells by Western blot analyses. Total levels of Cx43 and Panx1 increased after treatments with TNF-*α* plus ATP, TNF-*α*/IFN-*γ* or TNF-*α*/IL-1*β*, which caused the maximal effect on gap junctional communication (Figure 7(c)). Only the increase in total Cx43 levels was prevented by IL-6 in the same conditions that prevented the induction of dye coupling. Even when IL-6 prevented the increase in total Panx1 levels after treatment with TNF-*α*/IFN-*γ*, or TNF-*α*/IL-1*β*, coapplication of IL-6 failed to prevent the increase observed after TNF-*α* plus ATP treatment (Figure 7(c)).

## 4. Discussion

In this study, we demonstrated that extracellular ATP is required and advances the TNF-*α*/IFN-*γ*-induced dye coupling in cultured microglia, in an IL-1*β*-dependent manner. TNF-*α*/IFN-*γ*, but not TNF-*α* plus ATP enhances the basal and ATP-induced membrane permeability mediated by HCs. The increase in dye coupling induced by TNF-*α*/IFN-*γ* or TNF-*α* plus ATP was blocked by IL-6. Furthermore, inhibition of HCs prevents the pro-inflammatory molecules-induced upregulation of GJCs.

The ATP effects on the TNF-*α*/IFN-*γ*-induced dye coupling could be explained by activation of P2X receptors via ATP release, because the TNF-*α*/IFN-*γ*-induced dye coupling was prevented by oATP, a P2X receptor blocker. Activation of P2X receptors in microglia rises the [Ca^2+^]_*i*_ [1], which is known to induce gap junctional communication between cultured microglia in a PKC-dependent manner [24]. In agreement with the latter, BAPTA loaded microglia did not present dye coupling after treatment with TNF-*α* plus ATP. Thus, it is suggested that rises in [Ca^2+^]_*i*_ together with other downstream pathways contribute to up-regulate Cx43 levels and formation of HCs and GJCs as observed in other cell types [45, 67]. In HeLa cells expressing Cx43, rises in [Ca^2+^]_*i*_ enhance the cell surface levels of Cx43 HCs [45], a response that is directly associated to ATP release [68]. Thus, rises in [Ca^2+^]_*i*_ might contribute to increase the number of HCs in the plasma membrane of microglia. The increase in [Ca^2+^]_*i*_ could be initially mediated by activation of P2X receptors, but later on HCs might also contribute to increase their own activity favoring the Ca^2+^ influx because they are permeable to Ca^2+^ [69–71].

The cytokine-dependent induction of gap junctional communication between microglial cells was transient, as previously observed in dendritic cells and monocytes/macrophages [50, 51, 72]. The transient response might be explained by the production and release of anti-inflammatory cytokines, such as IL-6, IL-10, and TGF-*β*, by activated microglia [1]. Accordingly, IL-6 drastically reduces the cytokine-induced dye coupling between microglia treated with TNF-*α* plus ATP or TNF-*α*/IFN-*γ* as it also occurs in dendritic cells treated with TNF-*α*/IL-1*β* [50]. Since IL-6 reduces cell adhesion in breast cancer cells [73], a similar mechanism might affect the stability of cellular contacts between microglia, impairing gap junctional communication. In addition, IL-6 was found to prevent the rise in  [Ca^2+^]_*i*_. This might explain the inhibition of TNF-*α* plus ATP, because IL-6 did not prevent the increase in Panx1 levels. Although, IFN-*γ* signaling positively regulates purinergic receptors in microglia [11, 74], this might not explain the increase in dye coupling induced by TNF-*α*/IFN-*γ* because we found that IFN-*γ* delayed the appearance of dye coupling induced by TNF-*α* plus ATP. Further studies are required to unveil the mechanism underlying this cellular response.

We also found that in addition to TNF-*α*/IFN-*γ*, extracellular ATP and IL-1*β* also positively modulate the formation of GJCs in microglia. The link between purinergic signaling and IL-1*β* release has been well established in microglia [75], and here it was corroborated in EOC20 cells using IL-1ra, which prevented IL-1*β* release and establishment of dye coupling upon treatment with TNF-*α* plus ATP or TNF-*α*/IFN-*γ*. Interestingly, pro-inflammatory-like conditions (TNF-*α*/IL-1*β* or supernatant of microglia pretreated with LPS) increase HC activity but decrease gap junctional communication in primary astrocytes cultures [38]. However, we observed that TNF-*α*/IFN-*γ* increases both HC and GJC activity in microglia, indicating that different mechanisms control the functional expression of these channels in astrocytes and microglia. 

As shown in this work, the activity of microglial Cx and Panx HCs was increased by TNF-*α*/IFN-*γ*. Interestingly, Panx1 HCs and several Cx HCs are pathways of ATP release to the extracellular space in several cell types including astrocytes and microglia [25, 35, 37, 76, 77]. Therefore, enhanced HC opening may control ATP release from activated microglia maintaining a higher [Ca^2+^]_*i*_ compared with resting microglia [78]. Extracellular ATP could open Panx1 HCs, which are also activated after TNF-*α*/IFN-*γ*, leading to release of IL-1*β* [31]. Because, the HC activity remains low after treatment with TNF-*α* plus ATP, even after acute application of ATP, we propose that under these conditions ATP released by microglia via HCs was not required to induce IL-1*β* release. The latter is consistent with the prevention of TNF-*α*/IFN-*γ*-, but not TNF-*α* plus ATP-induced dye coupling in EOC20 cells treated with ^10^Panx1, a Panx1 HC blocker. In addition, we speculate that after treatment with TNF-*α* plus ATP P2X receptors also contribute in a Panx1 HC-independent way, as it has been proposed to occur during microglial proliferation [79]. The role of Cx43 HCs in TNF-*α*/IFN-*γ*–induced dye coupling was confirmed using Cx43^(E2)^ antibody, a specific Cx43 HC blocker. However, this conclusion should be taken cautiously because it was recently shown that several hours after Cx43^(E2)^ antibody application, gap junctional communication is partially reduced [42]. 

Under control conditions microglial cells express low levels of Cxs [23, 24, 26–28]. Accordingly, in this study we detected low levels of Cx43 and also Panx1. However, brain damage or cytokine exposure promotes microglial activation, and under this condition they present elevated levels of Cx43 and become coupled through GJCs [23, 24, 27, 28]. Here we found that TNF-*α* in presence of IFN-*γ* upregulates Cx43 GJCs in microglia as it was previously demonstrated [23, 28]. In addition, and similar to dendritic cells [50], TNF-*α*/IL-1*β* increased Cx43 levels in microglia. On the other hand, IL-6 prevents the formation of GJCs induced by pro-inflammatory cytokines in dendritic cells [50]. Accordingly, we found that IL-6 efficiently prevented the pro-inflammatory molecules-induced increase in GJC and HC activity in microglia. This effect could be explained, at least in part, by prevention of Cx43 and Panx1 upregulation by IL-6 and prevention of IL-1*β* release.

**Figure 8:**
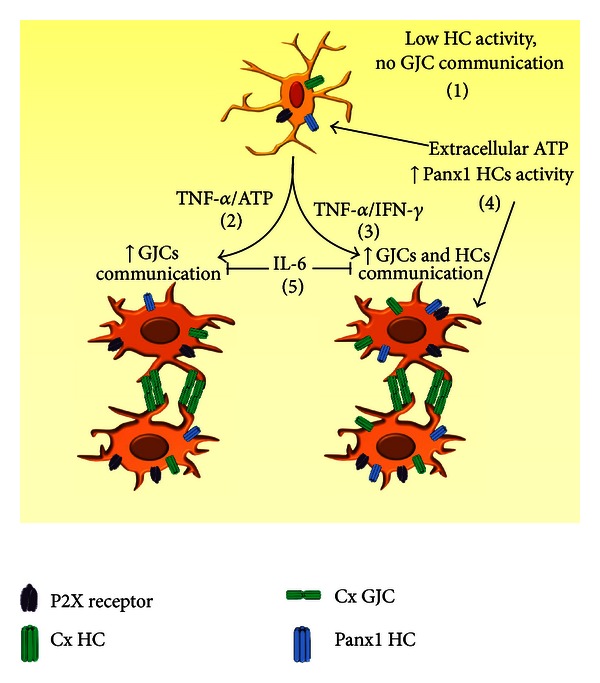
Cytokine-induced activation and the effect on gap junctional communication and HCs activity in cultured microglia. (1) Under resting condition, microglia express P2X receptors, Cx43, and Panx1, which have a low activity. Furthermore, no gap junction channel (GJC) communication is observed. (2) After TNF-*α* plus ATP exposition activated microglia exhibit gap junctional communication, but not intercellular communication mediated by hemichannels (HCs). (3) However, treatment with TNF-*α*/IFN-*γ* increased both GJC and HC functional state. (4) Extracellular ATP increases the Panx1 HC activity in both, resting or TNF-*α*/IFN-*γ*-activated microglia. (5) IL-1*β* release from activated microglia favors gap junctional communication. (6) IL-6 prevents IL-1*β* release and the increase in GJC and HC functional state.

So far, Panx1 has been demonstrated to form GJCs only in exogenous expression systems [71]. Together with the evidence that microglia from Cx43^del/del^ mice do not express functional GJCs [23] and that Cx43^(E2)^ antibody prevented the pro-inflammatory-induced dye coupling, it is suggested that dye coupling induced by TNF-*α* plus ATP or TNF-*α*/IFN-*γ* could be due to Cx43 GJCs. To recapitulate, we propose that in presence of extracellular ATP, Panx1 HC activity is enhanced and microglia migrate toward the injured site and release cytokines, as reported previously [33]. ATP could act in an autocrine and paracrine manner allowing IL-1*β* release and providing a pro-inflammatory microenvironment, which promotes an early up-regulation of Cx43 and Panx1, favoring the formation of HCs and GJCs in a stimulus-dependent manner (Figure 8). Later on, anti-inflammatory cytokines are produced and released to the extracellular milieu leading to reduction in intercellular communication mediated by HCs and GJCs similar to that of resting conditions. The latter is relevant because downregulation prevents a massive and/or prolonged ATP/glutamate release from microglia, which in turn can induce neurodegeneration [35, 56]. Thus, understanding the regulation of microglial purinergic receptors and intercellular communication via HCs and GJCs might contribute to modulate the timing of neuroinflammatory responses and led us to the identification of new therapeutic targets for neurodegenerative diseases [80].

## Supplementary Material

Short description for Supplemental Material: Microglia were seeded and after 48 h under control conditions were treated with 1 mM ATP or 1 ng/ml TNF-*α*, IFN-*γ*, IL-1*β* either alone or mixed. Cytokines were added simultaneously and ATP was added 2 h before measurement and is referred as cytokine(s) plus ATP. Treatment with IL-6, IL-1ra, oATP, La3+, Cx43(E2) antibody or 200 *µ*M 10Panx1 were simultaneous to cytokine treatment. We used *β*-GA for acute GJCs blocking.
