# Supplementary material for: ATP Is Required and Advances Cytokine-Induced Gap Junction Formation in Microglia In Vitro
Source: Mediators Inflamm. 2013 Apr 23;2013:216402. doi: 10.1155/2013/216402 (PMC3655668; doi:10.1155/2013/216402)
Supplement: Supplementary file 1 — Short description for Supplemental Material: Microglia were seeded and after 48 h under control conditions were treated with 1 mM ATP or 1 ng/ml TNF-α, IFN-γ, IL-1β either alone or mixed. Cytokines were added simultaneously and ATP was added 2 h before measurement and is referred as cytokine(s) plus ATP. Treatment with IL-6, IL-1ra, oATP, La3+, Cx43(E2) antibody or 200 µM 10Panx1 were simultaneous to cytokine treatment. We used β-GA for acute GJCs blocking. [file 216402.f1.pdf]

	Rat microglia		EOC20 cells		
**Treatment**	**I.D.C.**	**±SEM.**	**I.D.C.**	**±SEM**	**n**
Control - 3.5 h	5.5	1.6	5.1	1.4	6
ATP - 2 h	12,0	2,0	9.5	2.8	6
TNF-α - 3.5 h	13.3	3.3	12.4	2.8	3
TNF-α/ATP - 3.5 h	31.1	4.1	30.7	0.7	5
Control - 9 h	5.4	2.1	5.8	4.3	4
TNF-α - 9 h	N.D.	N.D.	9.7	0.2	4
IFN-γ - 9 h	N.D.	N.D.	17.5	4.8	4
IL-1β - 9 h	N.D	N.D	12.5	4.3	5
TNF-α/IFN-γ - 9 h	32.1	2.1	33.3	2.1	6
TNF-α/IL-1β - 9 h	N.D.	N.D.	32.5	2.5	4

Table S1 Incidence of dye coupling (I.D.C., raw data) in rat microglia and EOC20 cells under control conditions and after treatment with 1 mM ATP; 1 ng/ml TNF-α; 1 ng/ml IFN-γ; 1 ng/ml IL-1β and mixes as shown in the table and described in Methods. I.D.C. was calculated as the percentage of microinjected cells in which dye transference to neighbor cells was observed. N.D.: not determined.

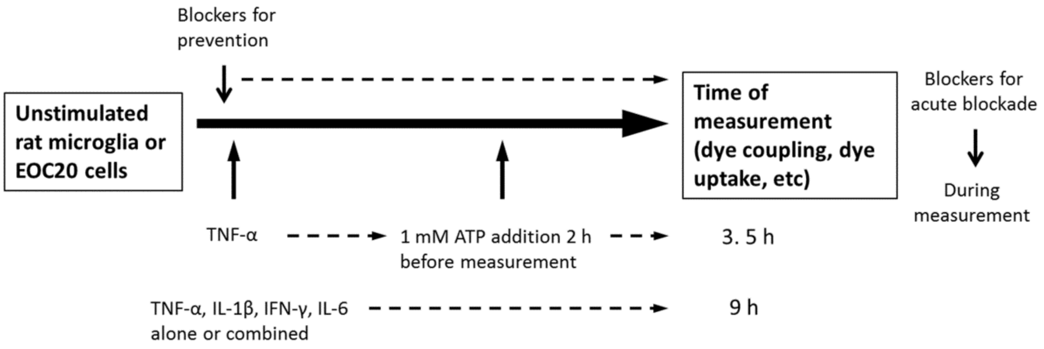

Figure S1. *Microglia treatment.* Schematic diagram illustrating the protocol used for microglia treatments with cytokines plus ATP and blockers.

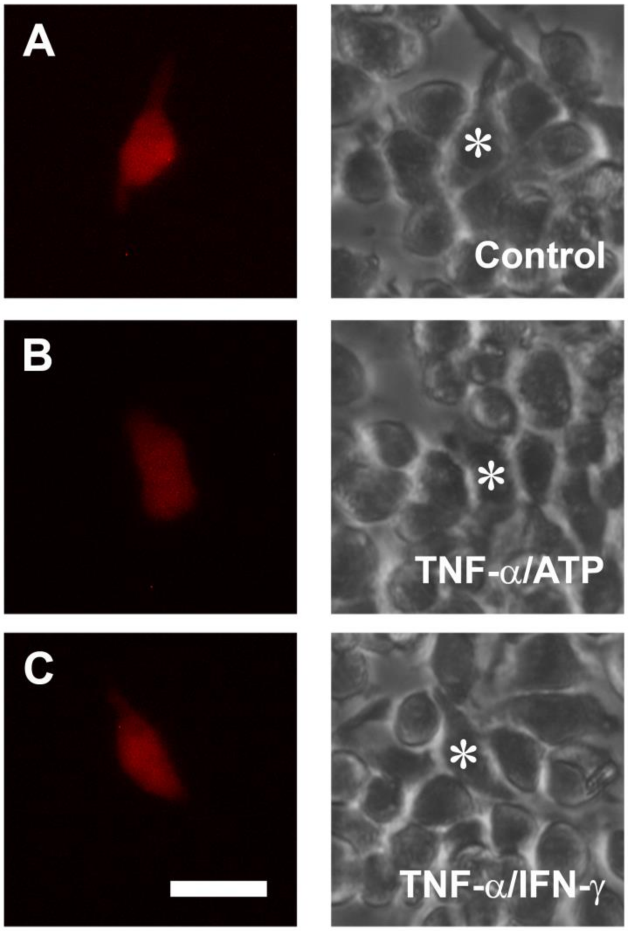

Figure S2. *Dye coupling induced by pro-inflammatory agents does not correspond to cytoplasmic bridges.* (A-C) Dye transfer was evaluated 5 min after rhodamine-dextran (RD, MW 10 kDa) microinjection of a single cell (indicated with an asterisk) in EOC20 cultures (A) under control conditions, or after treatment with (B) TNF-α plus ATP (TNF-α/ATP) for 3.5 h or with (C) TNF-α/IFN-γ for 9 h. Phase contrast views of each micrograph are shown in right panels. Scale bar: 20 μm.

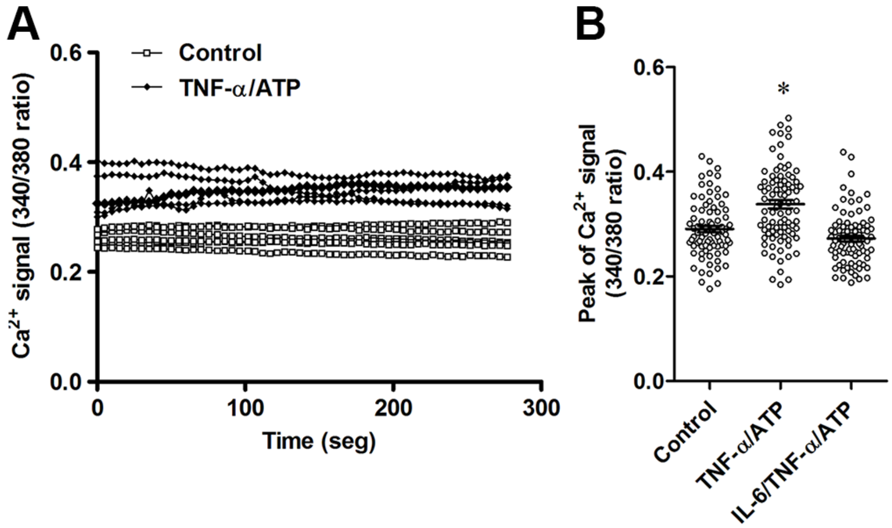

Figure S3. *IL-6 prevents the rise in $[Ca^{2+}]_i$ induced by TNF-α plus ATP.* (A) Graph showing the Ca^{2+} signal (340/380 ratio) in EOC20 cells loaded with Fura-2 in a time-lapse experiment under control conditions or treated 3.5 h with TNF-α plus ATP. Each line corresponds to one cell. (B) Graph showing the peak in Ca^{2+} signal in EOC20 cells under different conditions. Each dot corresponds to one cell, *P<0.05 versus control condition. Each line represents the mean ± SEM.

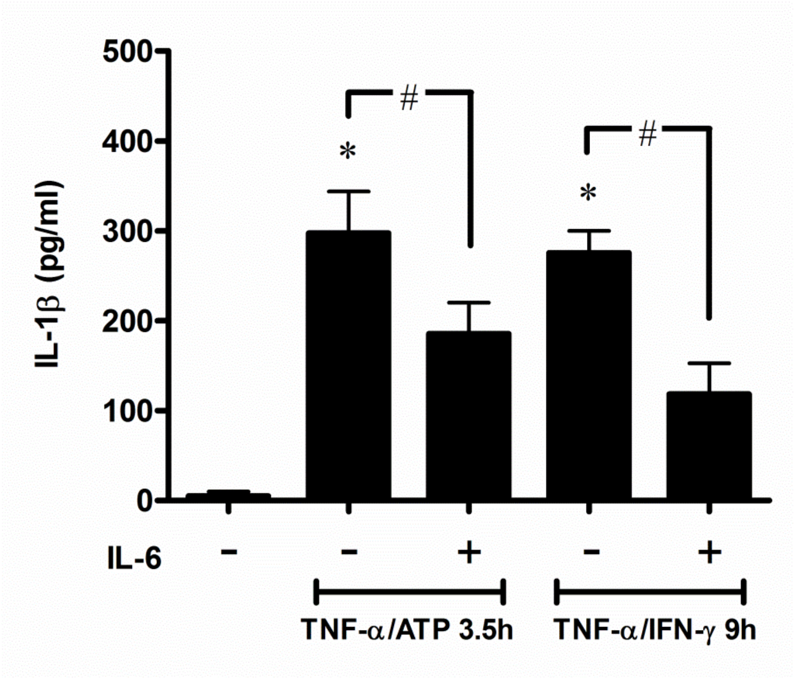

Figure S4. *IL-6 reduces pro-inflammatory-induced IL-1β release in EOC20 cells.* Graph showing IL-1β release from TNF-α plus ATP- or TNF-α/IFN-γ-treated EOC20 cells at times that induces dye coupling. *P<0.05 versus control condition, #P<0.05 between indicated treatments. Each bar represents the mean ± SEM.

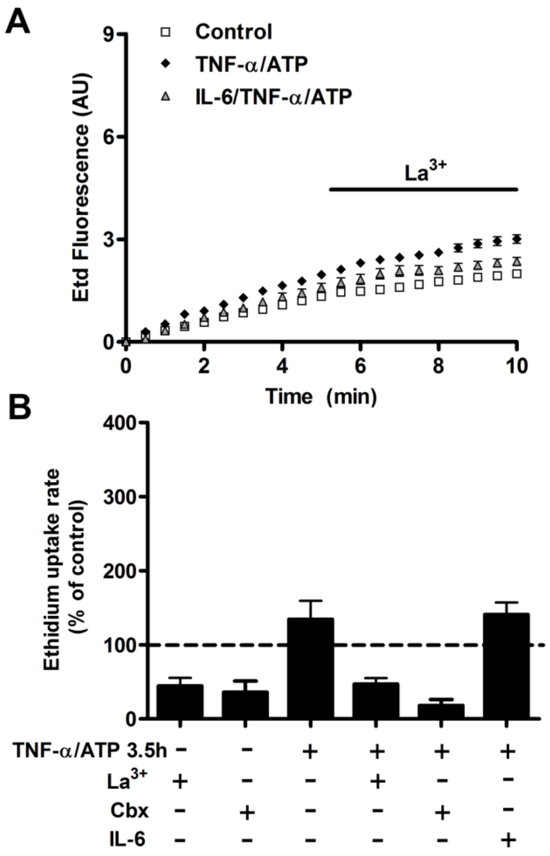

Figure S5. *TNF-α plus ATP did not increase membrane permeabilization in EOC20 cells.* (A) Time-lapse measurements of Ethidium (Etd) uptake in EOC20 cells under control conditions (white squares), or after treatment with TNF-α plus ATP (black diamonds) or IL-6/TNF-α plus ATP (gray triangles) for 3.5 h. Each value represents the mean ± SEM of 30 cells in each of 5 independent experiments. After 10 min of basal uptake, 200 µM La^{3+} was applied to the bath. (B) Ethidium (Etd) uptake rate expressed as percentage of Etd control conditions (dashed line) in EOC20 cells treated with TNF-α/ATP for 3.5 h. Is shown the acute effect of 200 µM La^{3+}, 10 µM carbenoxolone (Cbx), or pre-treatment with 10 ng/ml of interleukin-6 (IL-6) in TNF-α/ATP treated EOC20 cells. Each bar represents the mean ± SEM, n=5.

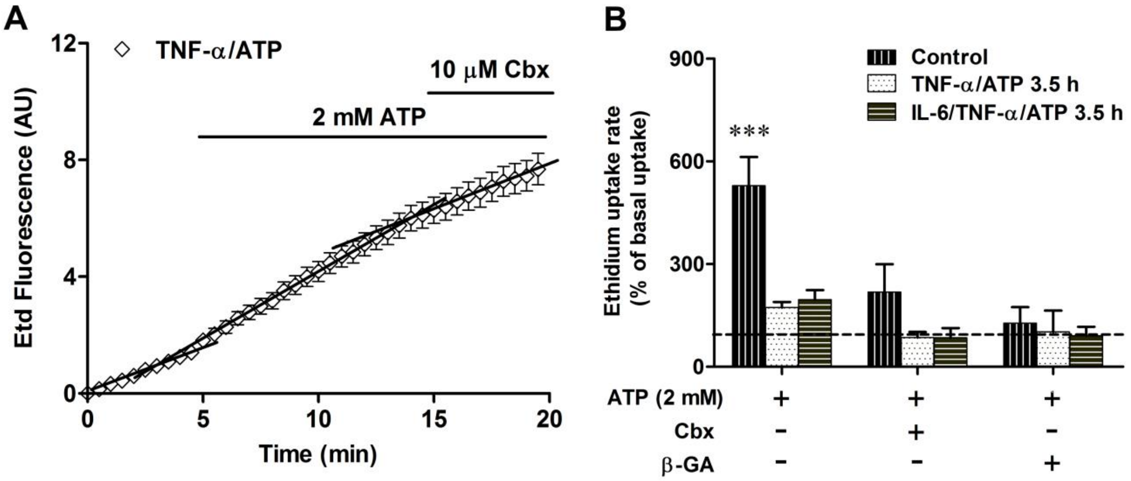

Figure S6. *Extracellular ATP did not increase the cell membrane permeability in EOC20 cells treated with TNF-α plus ATP.* (A) Time-lapse measurement. After 5 min of basal uptake, 2 mM ATP was added to extracellular solution. At 15 min of recording, 10 μM of carbenoxolone (Cbx) a HC blocker was added to the bath. Black lines denote the slope at different times of Etd uptake. Data represents the mean ± SEM of 30 cells in each of 5 independent experiments. (B) Graph showing the effect of acute application of extracellular ATP in EOC20 cells under control conditions or after treatment with TNF-α plus ATP, or with 10 ng/ml IL-6/TNF-α plus ATP for 3.5 h. The effect of acute blockade with 10 μM carbenoxolone (Cbx) or 50 μM 18-β-glycyrrhetinic acid (β-GA) is also shown. Data was normalized to basal uptake in each condition (dashed line) and represents the mean ± SEM. ***P<0.001 versus control condition.

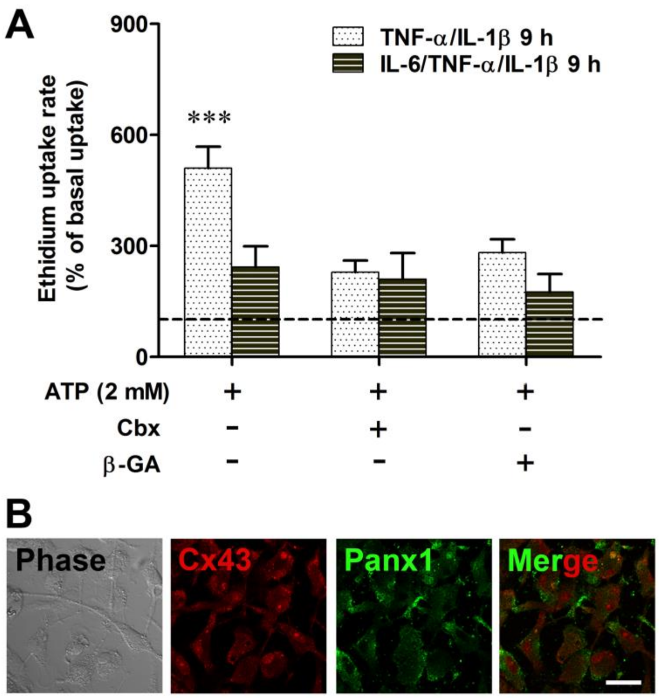

Figure S7. *TNF-α/IL-1β up-regulate Cx43 and Panx1 in rat microglia.* (A) Phase contrast and confocal images showing immunoreactivity for Cx43 (red) and Panx1 (green) in EOC20 cells after treatment with TNF-α/IL-1β for 9 h. Scale bar: 20 μm. (B) ATP-induced Etd uptake by EOC20 cells treated with TNF-α/IL-1β with or without 10 ng/ml IL-6 for 9 h. The effect of 2 mM ATP, 10 μM carbenoxolone (Cbx) or 50 μM 18-β-glycyrrhetinic acid (β-GA, a HC blocker) are also shown. Values were normalized to basal uptake in each condition (dashed line) and are expressed as the mean ± SEM, n=5. ***P<0.001 versus control condition.
